# Dynamic Genome-Wide Transcription Profiling and Direct Target Genes of CmWC-1 Reveal Hierarchical Light Signal Transduction in *Cordyceps militaris*

**DOI:** 10.3390/jof8060624

**Published:** 2022-06-11

**Authors:** Jiaojiao Zhang, Fen Wang, Mengqian Liu, Mingjia Fu, Caihong Dong

**Affiliations:** 1 State Key Laboratory of Mycology, Institute of Microbiology, Chinese Academy of Sciences, Beijing 100101, China; zhangjj@tib.cas.cn (J.Z.); wangfen@im.ac.cn (F.W.); liumengqian1011@gmail.com (M.L.); 2 Tianjin Institute of Industrial Biotechnology, Chinese Academy of Sciences, Tianjin 300308, China; 3 University of Chinese Academy of Sciences, Beijing 100049, China; 4 College of Life Sciences, Jiangxi Normal University, Nanchang 330022, China; mingjiafu@126.com

**Keywords:** *Cordyceps militaris*, transcriptomics, light-responsive genes, metabolite biosynthetic gene, CmWC-1, ChIP-seq, target genes

## Abstract

Light is necessary for primordium differentiation and fruiting body development for most edible fungi; however, light perception and signal transduction have only been well studied in model fungi. In this study, a hierarchical network of transcriptional response to light in *Cordyceps militaris*, one of the edible fungi, has been described on a genome-wide scale using dynamic transcriptome analysis. It was shown that light regulated the transcript of 1722 genes, making up 18% of the whole genome of *C. militaris*. Analysis of light-responsive genes in *C. militaris* identified 4 categories: immediate-early, early, late, and continuous light-responsive genes, and the gene number increased distinctly with prolonged light exposure. Light-responsive genes with distinct functional categories showed specific time-dependent regulation. The target genes of CmWC-1, the most important photoreceptor, were revealed by ChIP-seq. A total of 270 significant peaks corresponding to 427 genes were identified to be directly regulated by CmWC-1, among which 143 genes respond to light. Based on 270 ChIP-seq peaks, the binding site for CmWC-1 was identified as AAATCAGACCAC/GTGGTCTGATTT, differing from the binding site by the homolog in *Neurospora crassa*. Elucidating the mechanisms of light perception and signal transduction will be helpful for further research on the fruiting body development in edible fungi.

## 1. Introduction

Light is a critical environmental factor for mushroom primordium differentiation, fruiting body development, and some metabolic pathways. The influence of light on mushrooms has been reported in many species. Light inhibits mycelial growth of *Tuber borchii* [1], regulates asexual development in *Coprinopsis cinerea* [2], initiates mushroom formation in *Schizophyllum commune* [3], promotes fruiting body development in *Sparassis latifolia* [4], and affects the amounts of bioactive components in *Flammulina velutipes* [5]. Blue light primarily induces pileus growth of *Lentinula* *edodes* [6], brown-film formation [7], affects the development of primordium, and the quality of the fruiting body directly [8].

However, photoreception and light signal transduction in mushrooms have been studied mainly on the clone of photoreceptor genes and transcript analysis. White Collar (WC)-1 and WC-2 are important for regulating transcription after exposure to blue light. *Tuber borchii* is responsive to blue light, of which, *Tbwc-1*, a gene homologous to *wc-1* of *Neurospora crassa*, was isolated and cloned from *T. borchii* [1]. The *dst1* gene, encoding a *wc-1* homolog [9], and *dst2* gene, encoding a protein with a putative flavin adenine dinucleotide (FAD) binding-4 domain [10], were cloned and characterized at the molecular level. Both Dst-1 and Dst-2 may work as photoreceptors for blue light in *C. cinerea*. The genome of *S. commune* contains homologous genes of *wc-1* and *wc-2* of *N. crassa* [3]. It was shown that inactivation of these genes in *S. commune* resulted in a blind phenotype [3]. The blue-light photoreceptor genes *Le.phrA* and *Le.phrB* have been cloned and light-induced expression patterns have been determined in *L. edodes* [11,12].

Using transcriptome analysis, light-induced genes and signaling pathways associated with brown film formation in *L. edodes* were evaluated [7]. In the recent study, candidate genes involved in the morphological development of primordium of *L. edodes* were identified using transcriptome analysis as well [6]. Furthermore, growth and development of stipe, pileus, and gill of *Pleurotus* *ostreatus* were reported to be enhanced by blue light while pileus’ growth was slightly inhibited by red light [13]. Through comparative transcriptomic analysis of the stipe, pileus, and gill under blue-light, red-light, and dark conditions, it was shown that blue light improved *P. ostreatus* fruiting body growth rate via enhancement of glycolysis and the pentose phosphate pathway [13]. Light influences fruiting body development in *Tolypocladium guangdongense* and may also affect several other metabolic processes [14]. GATA-TFs have been analyzed in *T. guangdongens* and seven TgGATAs were identified. Among them, transcripts of *TgGATA1*, *TgGATA5*, *TgGATA6*, and *TgGATA7* were induced by light [15]. Moreover, 54 C6-type genes were light regulated in *T. guangdongens* [14]. Undoubtedly, more comprehensive and systematic studies are needed to elucidate light signaling in mushrooms.

Light perception and signal transduction have been well studied in filamentous fungi *N. crassa* and *Aspergillus nidulans*. In *N. crassa*, light was involved in the induction of sporulation and protoperithecia development [16], positive phototropism of perithecial beaks [17], induction of carotenoids synthesis [18], and regulation of the circadian clock [19,20]. Blue light-induced transcription in *N. crassa* is regulated by WC-1 and WC-2. VVD is the other blue light photoreceptor that has been implicated in light sensing in *N. crassa* [21,22]. WC-1 interacts with WC-2 to form a heterodimeric complex (WCC) to initiate light responses and VVD modulates the responses. The molecular mechanisms of light-mediated responses in *N. crassa* have been elucidated genome-widely in the past few years. Using large-scale quantitative analysis of microarrays with full-genome coverage, a catalog of 314 genes showing strong early- or late-light responses was provided in *N. crassa* [23]. The transcriptional response of *N. crassa* to light was studied using RNA-seq and the results showed that 532 genes (5% of all predicted genes) were light regulated [24]. Through functional profiling in *N. crassa*, 312 transcription factor genes, corresponding to 3.2% of the protein-coding genes in the genome, were identified [25]. Furthermore, chromatin immunoprecipitation (ChIP) sequencing was used to uncover direct targets of WCC in *N. crassa*. In response to light, WCC controls a transcriptional network that regulates 20% of all genes, binds to hundreds of genomic regions, and directly controls the expression of 24 transcription factor genes [26]. In *A. nidulans*, asexual conidiophores are produced in light, and durable ascospores are formed in the dark [27]. Light regulates the balance between sexual (cleistothecia formation) and asexual development (conidiation) and secondary metabolism in *A. nidulans* [28]. Snapshots of transcript and metabolite profiles during fungal development in dark or light were compared. The results showed that 2014 genes corresponding to 19% of the genome were expressed differentially when submerged vegetative hyphae being compared to surface development [29].

*Cordyceps militaris*, a well-known edible and medicinal fungus, has been used for a long history as a healthy food and traditional Chinese Medicine in China, Korea, and other East Asian countries [30,31]. It contains a wide range of bioactive compounds, such as cordycepin [32], carotenoids [33], beauveriolide [34,35], and pentostatin [36]. *C. militaris* has been demonstrated to have multiple biological and pharmacological activities, including anti-tumor [37], anti-inflammation [38,39], anti-oxidation [40], and antimicrobial functions [41]. In recent years, *C. militaris* has been regarded as a potential industrial mushroom.

Light is necessary for primordium formation, fruiting body development, and carotenoid production in *C. militaris* [42] and is able to promote conidiation and cordycepin production [43,44]. When *C. militaris* was exposed to light, the color of the mycelium changed from white to yellow or orange, and subsequently, the primordia begin to develop. No stromata were produced under darkness [42]. A series of studies on the light perception of this mushroom have been performed by our team. Blue light receptors CmWC-1, CmCRY-DASH, and CmVVD regulate fruiting body development and carotenoid production in *C. militaris*. *Cmwc-1*, a homologous gene of *N. crassa wc-1*, was cloned from the genome of *C. militaris* [42]. Inactivation of *Cmwc-1* resulted in thicker aerial hyphae, disordered fruiting body development, a significant reduction in conidiation, and carotenoid and cordycepin production [45]. CmCRY-DASH, consisting of DNA photolyase and flavin adenine dinucleotide binding domains, is another blue light receptor in *C. militaris*. Different from *Cmwc-1*, more carotenoids and cordycepin accumulated, and primordia were able to form, but the fruiting bodies were unable to elongate normally in the *Cmcry-DASH* inactivation strain [46]. Furthermore, gene deletion of the *Cmvvd*, a gene encoding for another blue light receptor, resulted in abnormal fruiting body development and a significant increment in carotenoid production [47].

The influence of light on growth, fruiting body development, and carotenoid accumulation of *C. militaris* as well as on its light transduction at the molecular level has been studied for many years. Here, a hierarchical network of transcriptional responses to light in *C. militaris* on a genome-wide scale using dynamic transcriptome analysis has been described. Direct targets of CmWC-1 were identified through ChIP-seq analysis. This study will be helpful for revealing the mechanism of light signaling transduction in mushrooms, and, more importantly, revealing the regulation of light on fruiting body development and secondary metabolite production in mushrooms.

## 2. Materials and Methods

### 2.1. Collection of Fungal Mycelia after Light Exposure for RNA-Seq 

The *C. militaris* strain CGMCC 3.16322 used in this study was maintained on potato dextrose agar (PDA) at 4 °C as a stock. The strain was incubated on the same medium in a Petri dish at 20 °C for two weeks in the dark before being used. For RNA-seq, a 9 mm disc was punched with a sterilized cutter from the inoculum and transferred to a fresh Petri dish containing the same medium topped with a layer of cellophane. After being cultured at 20 °C for 14 days in the dark, mycelia were collected from six plates and divided into two parts for subsequent RNA extraction. This group was designated as WTD. Meanwhile, the other plates were exposed to white light (1750 Lux) for 15 min (WTL15), 1 h (WTL1h), and 4 d (WTL4d) until the mycelia turned orange completely. Mycelia were collected as above and stored at −80 °C.

### 2.2. RNA Extraction, RNA Quality Examination, Library Preparation for RNA Sequencing and Library Examination

The total RNA was extracted using the E.Z.N.A. TM Plant RNA Kit (Omega, Stamford, CT, USA). The purity of the samples was determined by NanoPhotometer^®^ (IMPLEN, Westlake Village, CA, USA). The concentration and integrity of RNA samples were detected by an Agilent 2100 RNA nano 6000 assay kit (Agilent Technologies, Carpinteria, CA, USA).

A total amount of 1–3 μg RNA per sample was used as input material for the RNA sample preparations. Sequencing libraries were generated using the VAHTS Universal V6 RNA-seq Library Prep Kit for Illumina^®^ (NR604-01/02, San Diego, CA, USA) following the manufacturer’s recommendations and index codes were added to attribute sequences to each sample. Briefly, mRNA was purified from total RNA using poly-T oligo-attached magnetic beads. Then, fragmentation buffer was added to break the mRNA into short fragments. First-strand cDNA was synthesized using random hexamer primer and RNase H. Second-strand cDNA synthesis was subsequently performed using buffer, dNTPs, DNA polymerase I, and RNase H. Next, the double-stranded cDNA was purified by AMPure P beads or QiaQuick PCR kit. The purified double-stranded cDNA was repaired at the end, added with a tail, and connected to the sequencing connector; subsequently, the fragment size was selected, and finally, the final cDNA library was obtained by PCR enrichment

RNA concentration of the library was measured using the Qubit^®^ RNA Assay Kit in Qubit^®^ 3.0 to preliminary quantify and then dilute to 1 ng/μL. Insert size was assessed using the Agilent Bioanalyzer 2100 system (Agilent Technologies, Carpinteria, CA, USA). After the insert size met the expected size, the Bio-Rad CFX 96 fluorescence quantitative PCR instrument was used to accurately quantify the library effective concentration (Library effective concentration > 10 nm), and the reagent used was Bio-Rad kit iQ SYBR GRN.

### 2.3. Library Clustering and Sequencing

The clustering of the index-coded samples was performed on a cBot cluster generation system using the HiSeq PE Cluster Kit v4-cBot-HS (Illumina) according to the manufacturer’s instructions. After cluster generation, the libraries were sequenced on an Illumina platform Hiseq x ten and 150 bp paired-end reads were generated. A total of 8 libraries were constructed with two biological replicates for each sample.

### 2.4. Differential Expression Analysis 

Clean data were obtained by removing sequences containing adapters, poly-Ns, and low-quality reads from the raw data. The clean reads were mapped to the *C. militaris* genome [48] using TopHat v2.0.9 [49]. Differential expression analysis was performed using the Deseq2 tool [50]. The values in the matrix input were un-normalized counts of sequencing fragments. A corrected *p*-value of 0.001 and log2 (fold change) of 1 were set as the thresholds for significant differential expression, unless specified. A heat map was generated using the TBtools (Version 1.098696) [51]. Significantly enriched GO terms were clustered, summarized, and visualized using REVIGO [52].

### 2.5. Secondary Metabolite Biosynthetic Gene Clusters

Secondary metabolite biosynthetic gene clusters were predicted using antiSMASH version 5.1.0 (https://antismash.secondarymetabolites.org/, accessed on 25 October 2021) from *C. militaris* genome (AEVU00000000.1) [48]. Afterward, BLASTP (non-redundant protein sequences database, recovering the best 500 hits) (E-value < 1 × 10^−18^, identity ≥ 50%, coverage ≥ 55%, and matrix = BLOSUM62) was used to search and curate orthologous clusters among other filamentous fungi genomes. 

### 2.6. Transcription Factor

Fungi Transcription Factor Database (FTFD) [53] (http://ftfd.snu.ac.kr/index.php?a=view, accessed on 16 December 2021) was used to predict transcription factors in *C. militaris*. The FTFD pipeline is composed of four steps. Briefly, proteins carrying DNA-binding domains were collected from the CFGP data warehouse and classified as ‘Candidate TFs’ using 83 different InterPro terms. False positives were removed and ‘Putative TFs’ were classified. Then, known TFs that had not been properly annotated in the genome databases were manually added and tagged as ‘Characterized TFs’. Finally, all putative TFs and characterized TFs were analyzed phylogenetically.

### 2.7. Chromatin Immunoprecipitation (ChIP)

#### 2.7.1. Growth Conditions

For ChIP experiments, *Cordyceps militaris* wild-type strain CGMCC 3.16322 and *Cmwc-1* mutants which were constructed by Yang et al. in our lab [45] were grown in 150 mL of liquid media (containing 200 g/L potato, 20 g/L glucose, 3 g/L peptone, 1 g/L KH_2_PO_4_, 0.5 g/L MgSO_4_) in 500 mL shaking flask cultures at 150 rpm for 5 d in the dark. Two samples of mycelia for the later ChIP experiment were shaken in constant light (~1750 Lux) for 15 min.

#### 2.7.2. Cross-Linking

Samples were cross-linked in constant light and dark separately with 4.2 mL 1% formaldehyde for 15 min. Crosslinking was stopped by adding 6 mL of 2.5 M glycine and continued shaking incubation for 5 min. Samples were collected by filtration with 2 layers of gauze, rinsed with phosphate-buffered saline, dried with filter papers and mycelia were transferred to a 15 mL pre-cooled centrifuge tube, and frozen immediately with liquid nitrogen and stored at −80 °C.

#### 2.7.3. DNA Sonication

Frozen mycelia were ground to a fine powder in a chilled mortar and pestle with liquid nitrogen added. Briefly, 0.5 g powder was weighted and transferred to a new pre-cooled 15 mL centrifuge tube with 6 mL of ChIP lysis buffer (50 mM HEPES pH 7.5, 137 mM NaCl, 1 mM EDTA, 1% Triton X-100, 0.1% deoxycholate (Sigma D6750), 0.1% SDS, 1 mM PMSF, 1 μg/mL leupeptin, 1 μg/mL pepstatin A (Sigma, Burlington, MA, USA). Samples were vortexed and sonicated with Ultrasonic Homogenizer Scientz-IID (Scientz, Ningbo, China) with the following condition: 4 s ON and 4 s OFF at power level for a total of 20 min in a cold box. Tubes were centrifuged at 11,000 rpm for 15 min at 4 °C. Supernatants were transferred into new tubes. Five hundred microliters was reserved for reverse crosslinking to check sonication. DNA was extracted following the CTAB protocol and run on 1% agarose gel and fragment sizes ranged from 200 bp to 500 bp.

#### 2.7.4. Chromatin Immunoprecipitation

Protein-G beads were stored in 20% ethanol at −20 °C. One milliliter of beads was centrifuged in a 1.5 mL microfuge tube at 3000 rpm for 1 min at 4 °C and washed twice with 1 mL of lysis buffer. Then, 600 μL of lysis buffer with 12 μL 10 mg/mL BSA and 12 μL 100 mg/mL ssDNA was added to the washed beads and the beads were rotated at 4 °C overnight to coat beads. Protein concentration was determined from sonicated samples and 2 mg protein was taken for the later ChIP experiment. Ten microliters of protein sample added 490 μL lysis buffer was reserved as input and stored at −20 °C. Three microliters of CmWC-2 antibody (polyclonal antibody raised to a WC-2 protein fragment expressed in *Escherichia coli*) was added to each sample which was incubated at 4 °C with slow rotation overnight. The partial sequence of *Cmwc-2* (394–1179 bp) was introduced into plasmid pET-41a (+) and transformed into BL21 (DE3). The fusion proteins after purification were used as antigens for the preparation of antibodies to anti-CmWC-2. 

After incubation, 40 μL of beads was added to the samples. Samples were incubated for 4 h at 4 °C with rotation. Samples were washed with 1 mL of lysis buffer as above, then washed as above with 1 mL of wash buffer 1 (500 mL lysis buffer, 0.5 M NaCl), wash buffer 2 (1×LNDET; 0.25 M LiCl, 1% NP40 (Nonidet P40 Substitute, USB), 1% deoxycholate, 1 mM EDTA, 10 mM Tris, pH 8.0), and wash buffer 3 (50 mM Tris-HCl, pH 8.0; 1 mM EDTA), with 5 min of slow rotation at 4 °C for wash buffer 1 and wash buffer 2 while with 15 min of slow rotation at room temperature for wash buffer 3. After removal of the last wash, DNA was eluted from antibody with 300 μL of fresh elution buffer (1% SDS, 0.1 M NaHCO_3_) and the supernatant was transferred to a new tube. A second elution with 200 μL of elution buffer was performed so that the final elution volume was 500 μL. All samples were incubated overnight at 65 °C for reverse crosslinking.

#### 2.7.5. DNA Purification for ChIP Samples

After reverse crosslinking, DNA was extracted following the CTAB protocol, with 120 μL ddH_2_O as the final volume. 

#### 2.7.6. ChIP Sequencing, Alignment, Peak Finding

Library construction and sequencing were performed by Illumina HiSeq 2000 at Shenzhen Acegene Technology Co., Ltd. (Shenzhen, China). Briefly, the ChIPed DNA fragments were end-repaired, 5′-phosphorylated, 3′-dA-tailed, and ligated to adapters using the Axegen DNA Library Prep Kit (Axegen, Cat. No. AG0810) according to the manufacturer’s protocol. The adapter-ligated DNAs were purified and amplified by twelve cycles of PCR using Illumina 8 bp dual index primers. The constructed libraries were then analyzed by an Agilent 2100 Bioanalyzer and finally sequenced on Illumina platforms using a 150 × 2 paired-end sequencing protocol.

Paired-end reads from ChIP-seq experiments were quality checked with FASTQC (v0.11.7). Reads were trimmed and cleaned of contaminating Illumina adaptors using Trimmomatic (v0.36) and aligned to the *C. militaris* CM01 genome (AEVU00000000.1) [48] using BWA (Burrows Wheeler Aligner, v0.7.17). The resulting bam files were used as input for peak calling and for plotting. Peaks were called using MACS (Model-based Analysis for ChIP Sequencing, v2.0.9) (the threshold value was −g 1.2 × 10^8^ −p 1 × 10^−5^ −m 10,100) (https://github.com/taoliu/MACS, accessed on 16 September 2021). Peak calling was performed with the wild-type ChIP-seq samples and input control samples using an FDR cut-off of 0.05. 

#### 2.7.7. Motif Discovery and Enrichment in CmWC-1 Regulated Genes

To find sequence motifs within ChIP-seq peaks, 100 bp centered on each of the peaks was selected. Multiple Em for Motif Elicitation (Meme) was run with the minimum and maximum motif widths of 4 and 12, the zoops model, and the x branch option. The resulting 11 bp motif was reported for 54 sites. 

### 2.8. Quantitative Real-Time PCR

RNA extracted for sequencing was also used as templates to conduct reverse PCR. RT-qPCR was conducted using a CFX Connect Real-Time System (Bio-Rad, Singapore). The 10 μL qPCR solutions contained 2 ng of cDNA, 0.1 μM primers, and 5 μL of SYBR Green Mix (vazyme, China). The *rpb1* gene (*CCM_05485*) was used as an internal standard [44]. Relative gene expression levels were calculated using the 2^−ΔΔCt^ method [54]. The obtained data represented three biological replicates, with two technical replicates each. All the primer sequences used in this study are listed in Table S1.

## 3. Results

### 3.1. Gene Transcript Profiles after Light Exposure in Cordyceps militaris

To evaluate light-regulated gene expression, we performed mRNA-seq of RNA extracted from mycelia under darkness (WTD), mycelia being exposed to light for 15 min (WTL15), 1h (WTL1h), and 4d (WTL4d). A total of 380.4 million raw reads were generated by Illumina paired-end sequencing. After cleaning and quality checks, 366.9 million clean reads were obtained, with an average of 45.9 million reads per replicate (Table S2). More than 86.24% of the reads within each replicate could be mapped to the *C. militaris* genome [48] (Table S2). The Q30 percentages for all the sequences (with an error probability of 0.01; a high-quality indicator) were over 94%. The Pearson’s correlation coefficient of gene transcript between replicates for each sample was more than 0.90 (Figure S1), indicating good repeatability.

**Figure 1:**
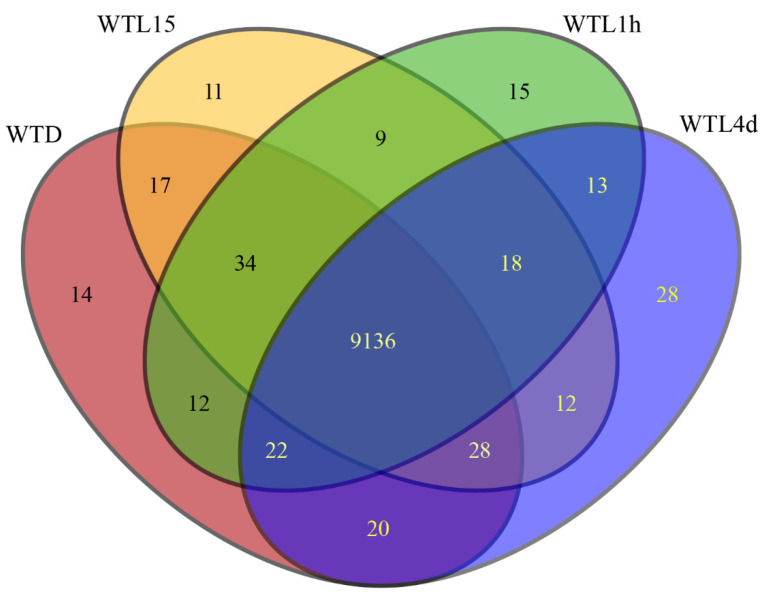
Venn diagram of transcribed genes in transcriptomes of *Cordyceps militaris* with different light exposure time.

The genome-wide distribution of gene transcription levels derived from the RNA-seq data is described in Figure S2. On a global scale, all genes could be divided into 4 categories according to their FPKM values, with the majority moderately transcribed (10 < FPKM ≤ 100) in all samples. Over 97% of genes were transcribed in all the tested 4 samples (Table S3). Among the 9651 genes of *C. militaris*, 9259 were transcribed in at least one sample and 9136 were transcribed in all samples (Figure 1). Briefly, 14, 11, 15, and 28 genes were transcribed uniquely in WTD, WTL15, WTL1h, and WTL4d, respectively (Figure 1).

### 3.2. Hierarchical Analysis of Light-Responsive Genes Identified in Cordyceps militaris

**Figure 2:**
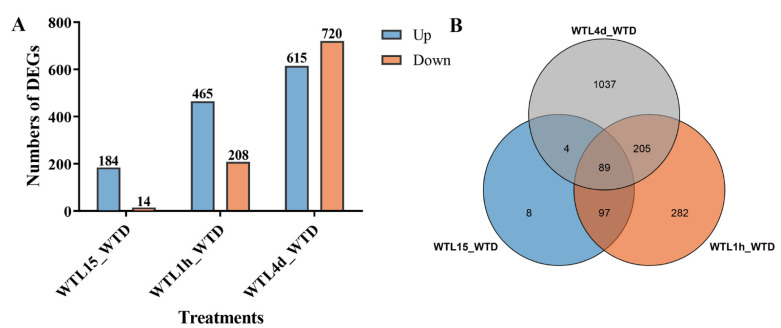
Analysis of DEGs between treatments with different light exposure time in WT strain. The number of genes differentially expressed after light exposure for 15 min, 1 h, and 4 d. Number of DEGs is indicated on the top of the histograms (**A**). Venn diagram of DEGs among the treatments with light exposure for 15 min, 1 h, and 4 d (**B**).

**Figure 3:**
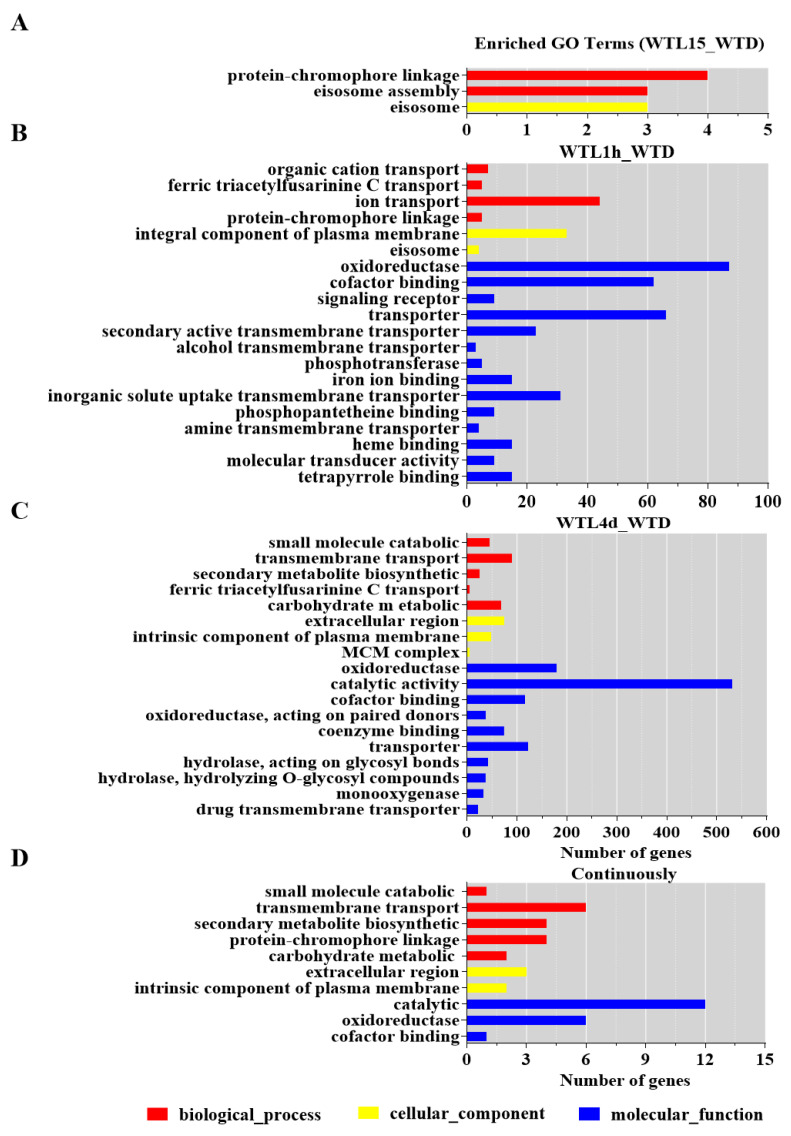
GO functional classification of differentially expressed genes. Enriched GO terms of immediate-early light-responsive genes (**A**), early light-responsive genes (**B**), late light-responsive genes (**C**), and continuous light-responsive genes (**D**). The significantly enriched GO terms (FDR < 0.05) removed redundancy using REVIGO were shown.

A systematic analysis using transcriptomics with full-genome coverage was performed to characterize light-inducible transcriptional changes in the wild-type strain (WT). Compared to darkness, there were 198 differentially expressed genes (DEGs) after being exposed to light for 15 min in the WT strain (WTL15_WTD) (onefold cut-off and FDR < 0.001) (Figure 2A), among which 184 genes were upregulated and 14 were downregulated. These genes were considered immediate-early light-responsive genes, only accounting for 2% of the *C. militaris* genome. Gene ontology (GO) analysis indicated that they were significantly enriched (FDR < 0.05) with eisosome (GO:0032126), protein-chromophore linkage (GO:0018298), and eisosome assembly (GO:0070941) (Figure 3A). 

Compared with darkness, 673 genes were transcribed differentially after light exposure for 1 h (WTL1h_WTD), of which 465 genes were upregulated and 208 were downregulated (Figure 2B). These genes were considered early light-responsive genes. GO analysis indicated that most of them were mainly enriched into molecular functions. The most significantly enriched GO terms were oxidoreductase activity (GO:0016491), cofactor binding (GO:0048037), signaling receptor activity (GO:0038023), integral component of plasma membrane (GO:0005887), and ion transport (GO:0006811) (Figure 3B, Table S4). The two significantly enriched GO terms of immediate-early responsive genes, eisosome (GO:0032126) and protein-chromophore linkage (GO:0018298), were also enriched.

Overall, 1335 genes were transcribed differentially after light exposure for 4 d when compared with darkness (WTL4d_WTD), which were considered the late-light responsive genes. Six hundred and fifteen genes were upregulated and 720 were downregulated (Figure 2A). Significantly enriched GO terms of these genes were also mainly molecular functions. The most significantly enriched GO terms were oxidoreductase activity (GO:0016491), catalytic activity (GO:0003824), cofactor binding (GO:0048037), extracellular region (GO:0005576), and small molecule catabolic process (GO:0044282) (Figure 3C, Table S4). Besides oxidoreductase activity, another three GO terms, cofactor binding, ferric triacetylfusarinine C transport (GO:0015686), and transporter activity (GO:0005215), were shared with early-responsive genes and there was no common enriched GO term with immediate-early responsive genes. In addition, secondary metabolite biosynthetic process (GO:0044550) and carbohydrate metabolic process (GO:0005975) were also significantly enriched.

Among all the light-responsive genes, a total of 1722 genes (17.84% of all the genes) were light regulated in the three light-exposed samples (Figure 2B, Table S4). Eighty-nine DEGs were shared in WTL15, WTL1h, and WTL4d, which were considered continuous light-responsive genes. The most significantly enriched GO terms of continuous light-responsive genes were extracellular region (GO:0005576), intrinsic component of membrane (GO:0031224), catalytic activity (GO:0003824), oxidoreductase activity (GO:0016491), and small molecule catabolic process (GO:0044282) (Figure 3D, Table S4).

### 3.3. Transcription Factors Responding to Light in Cordyceps militaris Were Mainly Zn2Cys6-Type and the Majority Responded to Light after Prolonged Exposure

**Figure 4:**
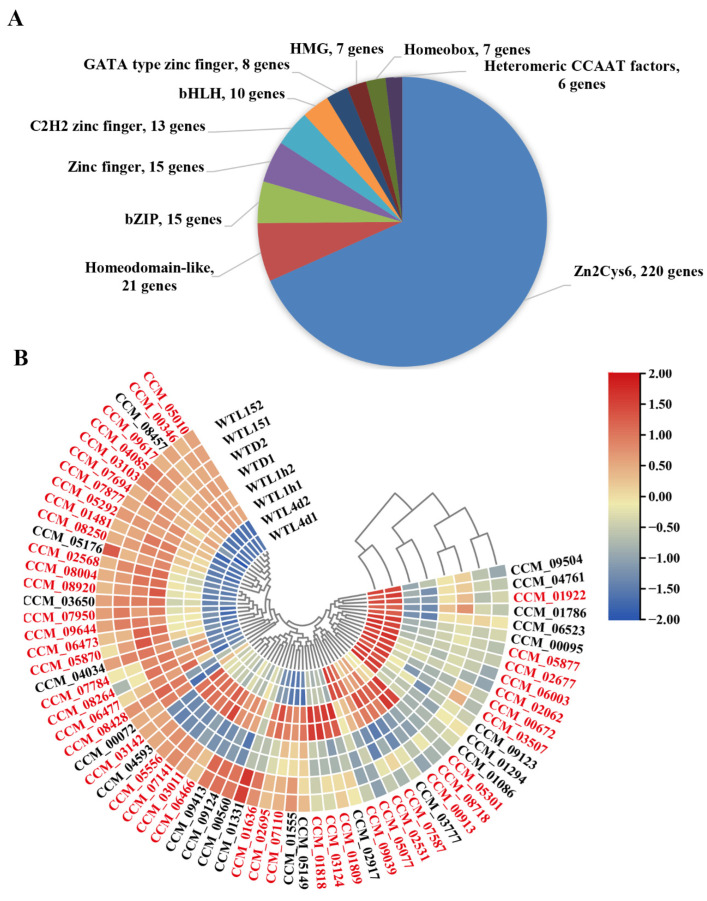
Distribution of transcription factors into major classes and heatmap for differentially expressed transcription factors in *Cordyceps militaris*. Each “slice” of the pie represents the class with a gene number of transcription factor more than 5. The number of transcription factor genes in each class is indicated (**A**). Each column represents expression values normalized by log2 (FPKM+1) at the indicated samples. Genes with similar patterns of expression are clustered together. The genes belonging to the Zn2Cys6 type have been marked with red (**B**).

Our previous research has revealed that CmWC-1 and CmVVD are key transcription factors (TFs) involved in photoreaction [42,47]. There were 358 TFs in total predicted by FTFD (fungal transcription factor database) [53] from the *C. militaris* CM01 genome, among which, 220 TFs belong to the Zn2Cys6 type (Figure 4A). The transcript profile showed that 68 TFs (19.0% of total TFs) can respond to light (Figure 4B). From the cluster analysis, WTD and WTL15 were grouped together, then grouped with WTL1h, whereas WTL4d was grouped alone (Figure 4B). It was indicated that most genes encoding TFs responded to light after prolonged exposure. 

Thirteen TF genes were immediate-early responsive and all of them showed up-regulation (Table S5). Among these TFs, CCM_01331 (a CP2-type TF) was the only one whose transcript level was significantly up-regulated over three-fold after light exposure for 15 min and then decreased to the same level as WTD, showing as photo adaption, the others were kept as early and late responsive. Twenty-seven TFs including CCM_00072 (CmWC-2) were early-responsive genes and only 5 TFs showed down-regulation with only about 1-fold. Fifty-one encoding genes of TFs belonged to late-responsive genes (Figure 4B, Table S5) and over half of them showed down-regulation. Tubby TF CCM_04593, HMG-type CCM_01555, and Zn2Cys6-type CCM_02531, 07141, and 06466 encoding genes belonged to continuous light-responsive genes and were induced at all the tested time points, suggesting that these TFs might function as key positive regulators constantly in the photoresponse.

Among the 68 DEGs, 46 genes were found to belong to Zn2Cys6 type TFs. It was indicated that TFs involved in the photoreaction in *C. militaris* were mainly the Zn2Cys6 type (Table S5), which was consistent with our previous report [45].

### 3.4. Metabolite Biosynthesis Cluster Regulated by Light Signal in Cordyceps militaris

**Figure 5:**
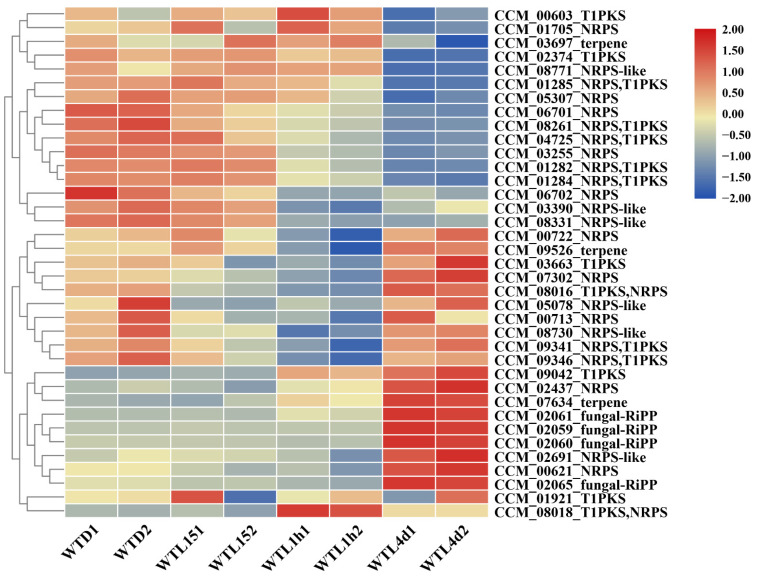
Heatmap of gene transcription of core genes involved in secondary metabolite cluster predicted by AntiSMASH under different light conditions. Each column represents expression values normalized by log2 (FPKM+1) at the indicated samples.

Thirty gene clusters, including 372 genes and 40 core genes, involved in the biosynthesis of secondary metabolites in *C. militaris* were predicted by AntiSMASH. The gene transcription profile of these 372 genes was analyzed from the transcriptome data (Table S6). Among the 40 core genes, the gene of *CCM_03050* (terpene) did not express, and *CCM_01518* (type I polyketide synthases, T1PKS) and *CCM_04722* (nonribosomal peptide synthetase (NRPS), T1PKs) transcribed very lowly with FPKM < 1 under all the tested conditions, while 16 core genes showed the highest transcription in the WT4d (Figure 5, Table S6). Sixteen core genes were late light-responsive genes, 10 were early light-responsive genes, whereas only 2 were immediate-early light-responsive genes (Figure 5). The results above indicated that the influence of light on metabolites mainly occurred in the late period, which was consistent with the GO analysis.

**Figure 6:**
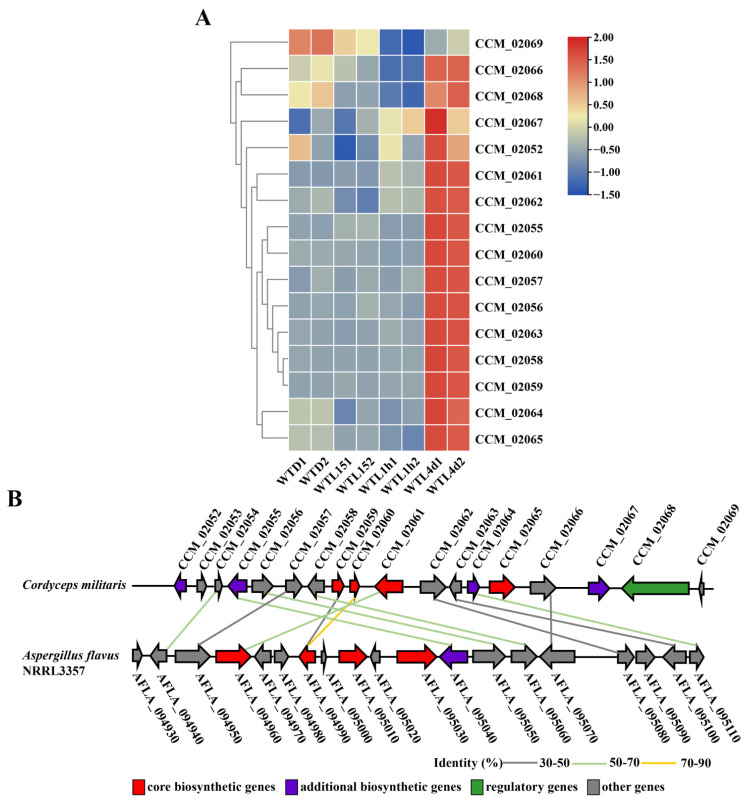
Gene expression and its homolog of the fungal-RIPP gene cluster compared with *Aspergillus flavus* in *Cordyceps militaris*. Gene expression of the fungal-RIPP gene cluster (**A**). Synteny analysis of gene cluster is responsible for ustiloxin biosynthesis in *Aspergillus flavus* and *Cordyceps militaris*. Horizontal arrows of the same color represent the homologous genes. The sequence identity between the homologous genes from two fungi is shown by bold solid lines with different colors (**B**).

The 4 core genes of one cluster of genes for a ribosomally synthesized and post-translationally modified (RiPP) metabolite (CCM-02059, 02060, 02061, and 02065) showed as the most up-regulated in WTL4d compared with WTD. This cluster contained 18 genes (CCM_02052-02069), in which CCM_02053 and 02054 did not express or expressed very lowly with FPKM < 1 under the 4 tested conditions and 15 genes showed the highest expression in the WT4d. Notably, the expressions of 11 genes were up-regulated significantly after light exposure for 4 d (Figure 6A) and most of them showed an up-regulation of over 6-fold. It was suggested that the transcript of this gene cluster is light induced. Blast analysis showed that three of the four core genes (CCM_02059, 02060, and 02061) have a similarity of over 50% (with the coverage over 50% and E value of <1 × 10^−18^ by BLASTP) with AFLA_094990 and AFLA_094960 in the gene cluster which has been confirmed to be responsible for the biosynthesis of ustiloxin B in *A. flavus* [55] (Figure 6B). Based on homologous analysis (Figure 6B), this cluster may be responsible for the biosynthesis of ustiloxin-related compounds in *C. militaris*, which is our ongoing project. 

Three core genes of one NRPS cluster (CCM_01282, 01284, and 01285) showed down-regulation in WT4d compared with WTD. A recent study has reported that this cluster is responsible for beauveriolides, the acyl-CoA:cholesterol acyltransferase inhibitor [35]. The biosynthesis of beauveriolides was regulated by light negatively.

Core gene CCM_09042 and another 2 genes CCM_09040 and 09041 in a T1PKS cluster showed significant up-regulation after light exposure for 1 h and 4 d. The expression of CCM_09042 encoding polyketide synthase has been demonstrated to be light induced in *C. militaris* (unpublished data). The researchers in our team are working on this cluster and the metabolite.

### 3.5. Light-Responsive Genes Regulated by CmWC-1 in Cordyceps militaris

CmWC-1 is a blue-light receptor and GATA-type TF [42]. The transcript of *Cmwc-1* is light induced [42]. Gene inactivation of *Cmwc-1* results in inhibited conidiation, disordered fruiting body development, and a significant reduction in carotenoid and cordycepin production [45], indicating *Cmwc-1* is essential for pigment formation and fruiting body development. A total of 1722 light-responsive genes were revealed on a genome-wide scale using dynamic transcriptome analysis in this study (Figure S3); however, not all the light-responsive genes were regulated by CmWC-1. In this study, the light-responsive genes regulated by *Cmwc-1* were uncovered based on our previous transcription data (PRJNA278309) of the WT and *ΔCmwc-1* strains under dark and light irradiation [45]. 

Firstly, compared to darkness, 943 genes were expressed differentially in WT after light exposure for 3 d (onefold cut-off and FDR < 0.001), of which 478 genes were up-regulated and 465 were downregulated. Then, expressions of these 943 DEGs were analyzed in the *ΔCmwc-1* strain. Eight hundred genes were found to be not expressed differentially anymore, 49 up-regulated genes turned into down-regulation and 40 down-regulated genes turned into up-regulation, indicating 889 light-responsive genes were regulated by *Cmwc-1*.

Among the 889 light-responsive genes regulated by *Cmwc-1*, 35 genes were TFs, indicating that 35 light-responsive TFs were regulated by *Cmwc-1* in *C. militaris*.

### 3.6. ChIP-Seq Analysis Identifies a Core Set of CmWC-1 Target Genes in Response to Light

To screen the target genes of CmWC-1 from genome-wide in *C. militaris*, ChIP-seq analysis using a CmWC-2-specific antibody after being exposed to light for 15 min was conducted as in our previous report [47]. ChIP-qPCR results showed that the negative control *Rpb1* had no obvious change in the WT and *ΔCmwc-1* strains under dark and light conditions. *Cmvvd* (CCM_04514) and *Cmfrq* (CCM_01014) which have been confirmed to be the target of CmWC-1 [47] showed significant enrichment in the WT strain under light compared with dark conditions, whereas they were not varied in the *ΔCmwc-1* strain under dark and light conditions [47], indicating that the ChIP sample for sequencing is qualified.

**Figure 7:**
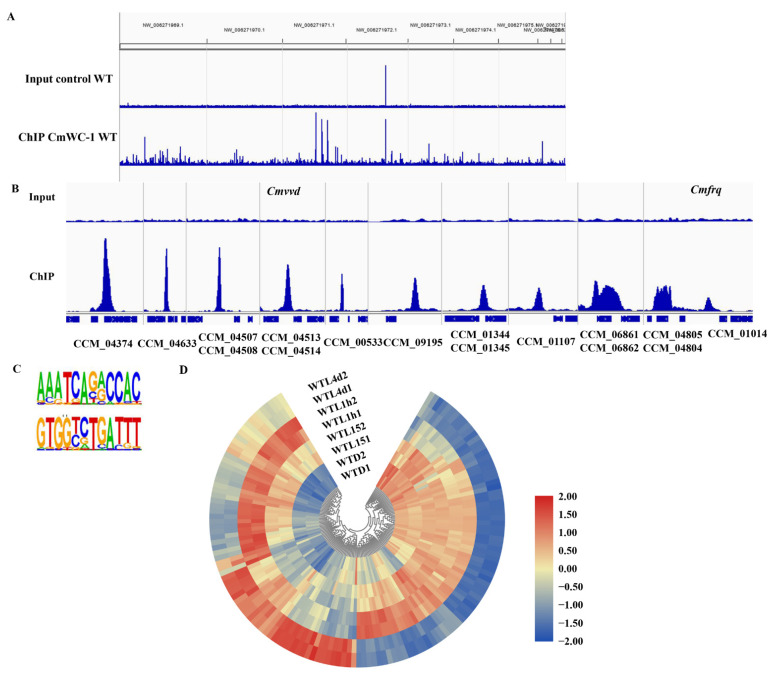
Genome-wide ChIP-seq analysis identified direct transcriptional targets of CmWC-1 in response to light for 15 min. Genome-scale view of ChIP-seq data for the ChIP sample. Blue lines rising above background are peaks identified as an excess of sequence fragments aligning to the genome of *Cordyceps militaris* strain CM01. The genome-wide view of the ChIP-seq reads aligned to the CM01 genome reveals strong visible peaks in the CmWC-2 antibody ChIP sample. Grey lines demarcate genome scaffolds (**A**). ChIP-seq peaks for selected genes including *Cmvvd* (CCM_04514) and *Cmfrq* (CCM_01014) (**B**). CmWC-1 binding motif identified using Homer (**C**). Gene expression of differentially expressed genes among the target genes of CmWC-1 obtained by ChIP-seq (**D**).

The overall numbers of Illumina reads used for peak calling of the ChIP-seq samples were 28,152,685 and 27,182,774 for ChIP and input control, respectively (Table S7). Reads were aligned to the *C. militaris* genome and a genome-scale view is shown in Figure 7A. A set of 357 peaks were identified and 270 of these peaks were located within the promoter regions, which corresponded to 427 genes (Table S7). 

Fifteen genes corresponding to the top 10 ChIP-Seq peaks are listed in Table S7. They were 2 heat shock proteins (CCM_04804 and 06821), 2 DNA repair proteins (CCM_04513 and 06822), 2 transcription factors (CCM_04514 and CCM_04374), an MFS transporter (CCM_04633), a mitochondrial protein (CCM_01345), a kinesin family protein (CCM_01344), and 6 hypothetical proteins (Table S7). UV is associated with light exposure in nature, which can explain the regulation by CmWC-1 of DNA repair genes. 

*Cmvvd* (CCM_04514) was among the 15 selected genes and has relatively high enrichment vs. input control (33-fold). *Cmfrq* (CCM_01014) also showed significant enrichment (9-fold), which was consistent with the result of ChIP-qPCR. Figure 7B shows examples of the peak regions from 16 target genes, including *Cmvvd* and *Cmfrq*. ChIP-qPCR confirmed that CmWC-1 could bind to the promoter regions of *Cmvvd* and *Cmfrq* in response to light [47].

A binding site for CmWC-1 based on 270 ChIP-seq peaks was identified as AAATCAGACCAC/GTGGTCTGATTT, a 12 bp region predominated by AAATCA in positions 1–6 and CCAC in positions 9–12 using Homer v4.9 [56]. Two positions were more variable, with a predominance of a guanine residue in position 7 and an adenine in position 8 (Figure 7C). 

**Figure 8:**
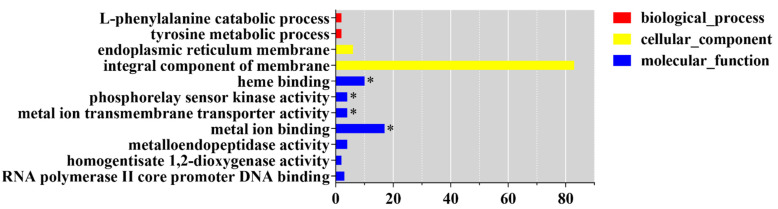
GO functional classification of CmWC-1 target genes identified by ChIP-seq. * Indicates significantly enriched GO terms (FDR < 0.05).

GO analysis showed that the most significantly enriched GO terms of CmWC-1 target genes were heme binding (GO:0020037), phosphorelay sensor kinase activity (GO:0000155), metal ion transmembrane transporter activity (GO:0046873), and metal ion binding (GO:0046872) (Figure 8). KEGG analysis showed that the most enriched KEGG terms were biosynthesis of antibiotics (cmt01130) and biosynthesis of secondary metabolites (cmt01110) (*p* < 0.05) (Table S8).

### 3.7. Light Response of Target Genes of CmWC-1 in Cordyceps militaris

The expression profile in the WT strain after light exposure of 427 target genes obtained by ChIP-seq was also analyzed. Among them, 143 genes can respond to light (Figure 7D, Table S9). Thirty-nine genes belong to immediate-early light-responsive genes, which were up-regulated after light exposure for 15 min. Seventy-three genes belong to early light-responsive genes, 66 of which were up-regulated after light exposure for 1 h and 7 were down-regulated. One hundred and four genes belong to late light-responsive genes, of which 46 were up-regulated and 58 were down-regulated after light exposure for 4 d. Twenty genes were continuous light-responsive genes, including photoreceptor genes CCM_00151 (*Phr1*) and CCM_04514 (*Cmvvd*), and frequency clock protein gene CCM_01014 (*Cmfrq*).

Among the 427 genes in which CmWC-1 binds to the promoter after light exposure for 15min, there were 20 transcription factors, including 11 Zn2Cys6-, 4 bZIP-, and 3 C2H2 zinc finger-type TFs (Table S9). According to the expression analysis, Zn2Cys6-type TFs *CCM_02531* and *07141* were continuous light-responsive genes and were up-regulated after light exposure for 15 min, 1 h, and 4 d. C2H2 zinc finger-type TF *CCM_01294* was a late responsive gene and was up-regulated after light exposure for 4 d. *CCM_05556* was up-regulated after light exposure for 15 min and 1 h. Notably, CmWC-1 was not included in the 20 TFs regulated by CmWC-1, indicating CmWC-1 was not regulated by itself or the CmWC-1/CmWC-2 complex, which was different from WC-1 in *N. crassa*, in which *wc-1* is directly regulated by the WCC in a positive feedback loop [26].

**Figure 9:**
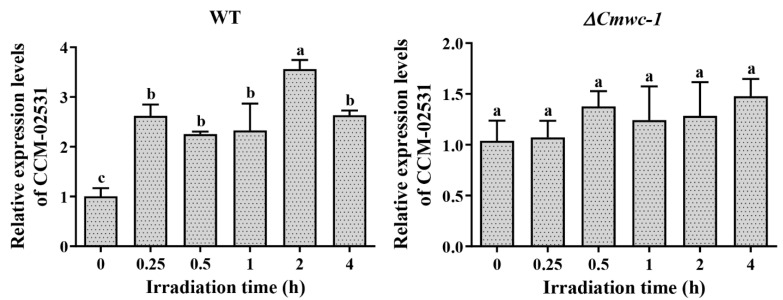
The expression levels of *CCM_02531* in the WT and *ΔCmwc-1* strains after light exposure revealed by qPCR. The relative expression levels of each gene in the different light exposure time were based on the standard levels of the light exposure time of 0 (under dark) in the WT and deletion mutants, respectively. Error bars indicate the standard deviations of biological replicates with two technical replicates. The different letters over the histogram indicate significant differences at *p* < 0.05.

In the target TF genes confirmed to be regulated by CmWC-1 directly, GATA TF CCM_04514 (*Cmvvd*), Zn2Cys6-type TFs CCM_05556 and 09159, and C2H2 zinc finger TF CCM_01294 were differentially expressed in the WT strain but not differentially expressed in the *ΔCmwc-1* strain, while up-regulated Zn2Cys6-type TFs CCM_02531 were shown to be down-regulated (Table S10). The result showed that 5 TFs CCM_04514, 02531, 01294, 05556, and 09159 were regulated directly by CmWC-1. The relative expressions of *CCM_02531* and *CCM_04514* were determined by qRT-PCR and the results showed that *CCM_02531* (Figure 9) and *CCM_04514* [47] were up-regulated after light exposure for 15 min compared with darkness in WT, while they lost photoreaction in *ΔCmwc-1*, indicating that the expressions of *CCM_02531* and *CCM_04514* were light inducible and dependent on CmWC-1. 

## 4. Discussion

Fungal photoreceptors and light signal perception have been described in recent years, and the complexity of the light-sensing system has emerged. However, a good understanding of signaling processes at the molecular level is limited to some model fungi, little is known about mushrooms. In this study, dynamic genome-wide expression analysis of light-responsive genes in *C. militaris* and the target genes of CmWC-1, the most important photoreceptor, revealed by ChIP-seq, provided novel insights into fungal light sensing and signaling pathways.

Our transcriptomic results showed that light regulates the expression of 1722 genes, making up 18% of the whole genome of *C. militaris* after exposure to light for 15 min, 1 h, and 4 d. Furthermore, 1615 genes, accounting for ~13.4% of the genes in the genome, were blue-light regulated in *Trichoderma guizhouense* through genome-wide gene expression [57]. The ratio of light-responsive genes in the genome of *C. militaris* may be overestimated because cultures under dark conditions for 14 d rather than 18 d were used to be compared with WTL4d. Among the 1335 DEGs of WTL4d_WTD, 298 DEGs were shared with WTL15_WTD and WTL1h_WTD, which were determined to be light-responsive genes. For the other 1037 DEGs, some growth-related genes may be included. It has been reported that the number of light-responsive genes ranged from 3% to 14% in the genome of *N. crassa* due to the use of different microarray platforms, strains, culture conditions, and statistical cut-offs [24]. Further studies should be conducted for a better understanding of gene regulation in *C. militaris* in response to light.

**Figure 10:**
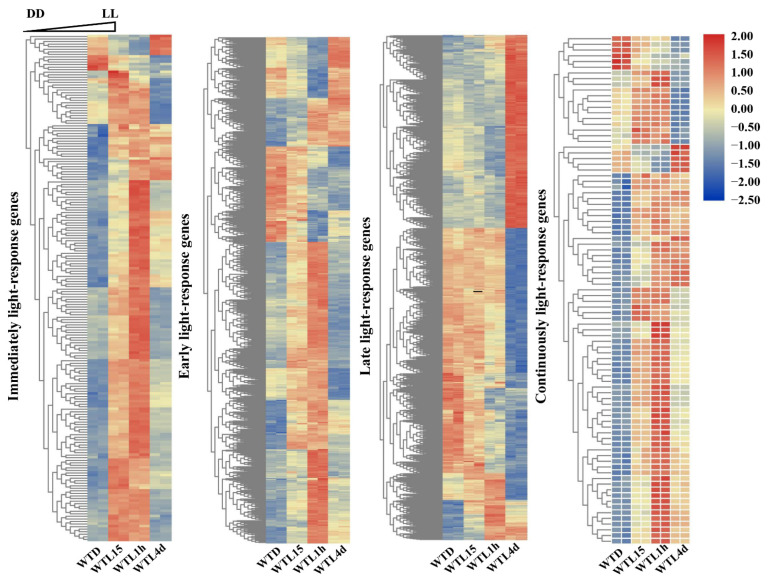
Classification of light-response genes. Immediately, early, late, and continuously light-responsive genes respond to light after exposure for 15 min, 1 h, 4 d, and always from 15 min to 4 d, respectively. For each lane, from left to right, the individual columns correspond to light treatment for 0, 15 min, 1 h, and 4 d, respectively.

Hierarchical analysis of light-inducible responses identifies early light-response genes responding to light after exposure for 15 and 45 min, and late light response genes responding to light between 45 and 90 min in *N. crassa* [23]. In the present study, hierarchical analysis of light-responsive genes in *C. militaris* identified four groups: immediate-early, early, late, and continuous light-responsive genes (Figure 10), which respond to light after exposure for 15 min, 1 h, 4 d, and always from 15 min to 4 d, respectively. The gene number increased distinctly with prolonged light exposure (198, 673, and 1335 genes for exposure for 15 min, 1 h, and 4 d, respectively) and only 89 genes were continuous light-responsive genes. The distinct kinetics of induction that distinguishes the immediate-early, early, late, and continuous light responses suggested that most of the light response genes are separately regulated while a part of them are coordinately regulated. There might be different molecular mechanisms underlying the timing control of each response.

**Figure 11:**
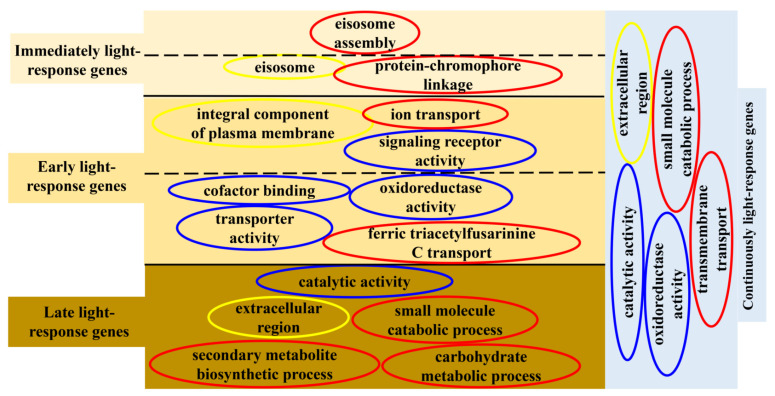
Gene-function clustering of immediately, early, late, and continuously light-responsive genes. Red box represents biological process, yellow box represents cellular component, and blue box represents molecular function. The dotted line represents the repeated GO terms between two adjacent light response periods.

Immediate-early light-responsive genes can be activated and transcribed within 15 min after light stimulation. GO analysis showed that these genes were involved in protein-chromophore linkage, eisosome, and eisosome assembly (Figure 11). Eisosomes are stable protein complexes at the plasma membrane of fungal cells which have been reported to have functions in a wide range of biological processes, including plasma membrane organization, cell wall synthesis, stress responses virulence, and so on [58]. In the phylogenetic close species *Beauveria bassiana*, eisosome proteins Pil1A, Pil1B, and Sur7 were found to be involved in multiple stress responses [59,60]. It was reported that photoreceptor PHR1 is involved in protein-chromophore linkage [61]. Therefore, it is speculated that after light exposure, photoreceptors bind to other proteins through protein-chromophore linkage, and *C. militairis* responds to oxidative pressure caused by a light signal through eisosomes and eisosomes assembly quickly. As a result, the immediate-early genes can respond to light and thus regulate the growth, conidiation, and metabolites production of *C. militairis*. 

Early light-responsive genes in *C. militairis* were involved in oxidoreductase activity, cofactor binding, signaling receptor activity, integral component of plasma membrane, ion transport, and also involved in eisosome and protein-chromophore linkage (Figure 11). In *N. crassa*, early light-responsive genes (15 and 45 min after onset of light) are involved in lipid, fatty acid, and isoprenoid metabolism, biosynthesis of vitamins, cofactors, and prosthetic groups, secondary metabolism, DNA processing, cellular signaling, photoperception and response, circadian rhythm, etc. [23]. Obviously, early light-responsive genes in *C. militaris* and *N. crassa* shared in photoperception, cellular signaling, and response to external stimuli. However, *N. crassa* initiated the secondary metabolism especially, whereas oxidoreductase activity is important for *C. militaris* in the early light response. It is especially puzzling in the case of eisosomes, since they also exist in organisms without any known photobiology, such as *S. cerevisiae*.

Late light-responsive genes in *C. militaris* were involved in oxidoreductase activity, catalytic activity, extracellular region, small molecule catabolic process, and carbohydrate metabolic process (Figure 11). In addition, secondary metabolite biosynthetic process was also significantly enriched (Figure 11), indicating that light-induced metabolite biosynthesis occurred. Late light-responsive genes were involved in carbohydrate metabolism, oxidation of fatty acids, and oxygen/radical detoxification reaction in *N. crassa* [23]. There was no shared GO term between late and immediate-early responsive genes in *C. militaris* (Figure 11), indicating the difference between late and immediate-early light response. 

Continuous light-responsive genes in *C. militaris* were involved in catalytic activity, oxidoreductase, and small molecule catabolic process (Figure 11). Those genes responding to continuous light may be involved in the regulation of enzyme activity in metabolic processes to control the production of metabolites. This could explain why some metabolites such as carotenoids and beauveriolides [35] are regulated by light. In summary, light-responsive genes with distinct functional categories show specific time-dependent regulation, illustrating a temporal sequence of important cellular events for *C. militaris* in response to daily light. Finally, for this classification, there are some overlapping of the different categories and each gene has only one response. They may differ not only in the onset of the response but also in the type of subsequent attenuation.

Given our previous finding that the light-responsive TF CmWC-1 is the primary and important component in mediating light signaling [45], we predicted that it might initiate a regulatory cascade leading to the activation of light responses through a transcriptional hierarchy. Our analysis showed that 68 TFs were light regulated, most of them responded to light lately and the majority of them were Zn2Cys6 type. The number of down-regulated TFs increased from 0 to 28, with prolonged light exposure time, indicating that most TFs were light induced and photoadaptation occurred also for TFs.

Twenty-four TF genes that have the potential to control downstream target genes on a second hierarchical level were direct targets of WCC in *Neurospora* [26]. SUB-1, an early light-responsive TF, is involved in regulating some early and most late light responses as WC-1 in *N. crassa* [23]. The gene encoding a homologous protein in *C. militaris* (CCM_00560) is immediate-early and early responsive. SUB-1 is believed to be required for efficient transduction of light signals to some early responsive and most late responsive genes under WCC control; however, the transcription of *CCM_00560* was independent of CmWC-1 in *C. militaris*. In this study, it was confirmed that five TFs, *CCM_04514*, *02531*, *01294*, *05556*, and *09159* were regulated directly by CmWC-1 and may control downstream target genes on a second hierarchical level in *C. militaris*.

Thirty gene clusters including 372 genes and 40 core genes were involved in the biosynthesis of secondary metabolites in *C. militaris*, of which 28 core genes were light responsive and most of them were late responsive. It was found that the biosynthesis of beauveriolides was inhibited after light exposure for over 1 h. However, most of the metabolites have not been identified until now. The fungal-RiPP cluster was predicted to synthesize ustiloxin-related compounds and the gene transcriptions of the cluster responded to light. Ustiloxin is a secondary metabolite known to be produced by *Ustilaginoidea virens* [55]. It was reported that ustiloxins not only exhibited toxicity in the liver and kidney of mice and in the early growth and development of zebrafish [62,63] but also functioned as phytotoxins to inhibit the elongation of radicle and induce the swelling of seedling root in rice [64]. Ustiloxins could strongly inhibit tubulin polymerization and depolymerize pre-formed microtubules in vitro, and these ustiloxins could bind the vinca domain (or RZX/MAY site) of tubulins [65,66]. The safety of *C. militaris* induced by ustiloxins should be a concern, which is our ongoing project. 

Further, light-induced transcriptional responses of *C. militaris* dependent on WC-1 on a genome-wide scale were further described here, and 889 light-responsive genes were regulated by CmWC-1 directly or indirectly. To identify direct targets of CmWC-1, we performed ChIP-seq with anti-WC-2 antibody subjected to 15 min light exposure. By ChIP-seq, 270 significant peaks corresponding to 427 genes were identified to be directly regulated by CmWC-1. Direct targets of the *N. crassa* WCC were uncovered using ChIP-seq with anti-WC-2 antibody subjected to an 8 min light pulse [26]. More than 400 significant regions of WCC enrichment were identified. Differently from RNA-seq, cultures in liquid medium were used for ChIP-seq. Since light does not pass equally well through liquid and solid media, some target genes regulated by CmWC-1 may not be screened completely in this study, which can explain the number of target genes regulated by CmWC-1 identified in ChIP-seq was lower than that in RNA-seq.

Target genes encoding classified proteins were summarized according to their functional categories with functions in the cell cycle, transcription, protein binding, response to the environment, and cellular components [26]. The biological functions of CmWC-1 target genes were heme binding, phosphorelay sensor kinase activity, metal ion transmembrane transport, and metal ion binding. It was reported that genes involved in metal ion homeostasis were needed for *N. crassa* to control metal ion homeostasis during exposure to light [24]. It was implied that target genes regulated by CmWC-1 in *C. militaris* function in a similar way to that of *N. crassa*.

A binding site for CmWC-1 based on 270 ChIP-seq peaks was identified as AAATCAGACCAC/GTGGTCTGATTT, a 12 bp region predominated by AAATCA in positions 1–6 and CCAC in positions 9–12. Two positions were more variable, with a predominance of a guanine residue in position 7 and an adenine in position 8. The results of ChIP-qPCR confirmed that *CCM_04514* (*Cmvvd*) and *CCM_01014* (*Cmfrq*) were the target genes of CmWC-1 [47] and the binding sites were identified in the promoter, respectively (Figure S4). The binding site for WCC in *N. crassa* was identified as GATCGA with variability in the first and last bases based on 1 kb regions centered on the WCC ChIP-seq peak at 29 genes that were confirmed to be light induced [26]. The SrbA-bound DNA motif in *A. fumigatus* was identified as an 11 bp binding region predominated by ATCA in positions 1–4, a cytosine in position 7, and an adenine in position 9. Other positions were more variable, with a predominance of cytosine residues [67].

In this study, an antibody against CmWC-2 was used for the ChIP experiment. We have also tried the CmWC-1 antibody. However, the bands were not clear and exhibited a mass of black when tested in Western blot using CmWC-1 antibody, which may be caused by the phosphorylation of CmWC-1. WC-2 antibody was used to reveal direct targets for *Neurospora* White Collar Complex [23]. It means that only the binding of the WC-1/WC-2 complex is being tracked and the genes to which CmWC-1 DNA binds on its own would be missing. In addition, genes that CmWC-2 bind to in the dark and their transcription after light exposure should be studied further for a better understanding of target genes regulated by CmWC-1. There are phenotype differences between mutants of the two homologs, e.g., *A. nidulans* [28], the light response of *Cordyceps* is similar to *Neurospora* while it is different from *A. nidulans*.

The expression profile of 427 target genes in WT and *ΔCmwc-1* strains after light exposure was also analyzed combined with the transcriptome data obtained by Yang et al. [45]. Compared with darkness, 69 genes can respond to light in WT, of which 66 of them were confirmed to be dependent on CmWC-1 since they lost or changed their photoreaction in the *ΔCmwc-1* strain. 

Five TFs *CCM_04514*, *02531*, *01294*, *05556*, and *09159* were regulated directly by CmWC-1. qRT-PCR confirmed that *CCM_02531* (Figure 9) and *CCM_04514* [47] were up-regulated after light exposure for 15 min compared with darkness in the WT strain, indicating both are immediate-early light-responsive genes. Whether all the genes with WC-1 binding sites should be enriched in immediate-early light-responsive genes has not been reported in the other species.

Our previous study revealed that gene disruption of *Cmwc-1* resulted in inhibited conidiation, disordered fruiting body development, and a significant reduction in carotenoid and cordycepin production [45], indicating *Cmwc-1* is necessary for pigment formation and fruiting body development. In this study, the light-responsive genes regulated by *Cmwc-1* were uncovered and revealed the hierarchical regulation of CmWC-1. However, it is necessary to verify the regulated pathway by experiments.

Fungi have evolved complex transcriptional networks that mediate developmental changes in response to light. In this study, the hierarchical network of the transcriptional response to light in *C. militaris* was found to be greatly different from the model fungi *N. crassa*. Light is an essential environmental factor for fruiting body development in most edible fungi. Elucidating the mechanisms of light perception and signal transduction will be helpful for research on fruiting body development in edible fungi.

## Data Availability

Raw data of RNA-seq and ChIP-seq were submitted to NCBI Sequence Read Archive (SRA, accessed on 22 April 2022) under BioProject PRJNA828810 and PRJNA830590.

## Supplementary Materials

The following supporting information can be downloaded at: https://www.mdpi.com/article/10.3390/jof8060624/s1, Figure S1: The Pearson’s correlation coefficient of gene transcript between replicates for WTD, WTL15, WTL1h, and WTL4d; Figure S2: The genome-wide distribution of gene transcription levels derived from the RNA-Seq data; Figure S3: Heatmap of light-responsive genes across all timing of light exposure and darkness based on their gene expression values in term of FPKMs; Figure S4: The binding sites of CmWC-1 identified in the promoter of *CCM_04514* (*Cmvvd*) and *CCM_01014* (*Cmfrq*). Table S1: Primers used in this study; Table S2: Quality evaluation of RNA-seq data; Table S3: Summarized results of expression genes revealed by RNA-seq; Table S4: Gene Ontology analysis of light-responsive genes belonging to immediate-early, early, late and continuous groups; Table S5: The expression profile of transcription factor genes after light exposure; Table S6: The expression profile of metabolite synthetic genes after light exposure; Table S7: The overview of ChIP-seq; Table S8: Gene ontology analysis of target genes of CmWC-1 revealed by ChIP-seq; Table S9: The expression profile of target genes of CmWC-1 obtained by ChIP-seq in the WT strain after light exposure; Table S10: The expression profile of target genes of CmWC-1 revealed by ChIP-seq in the *ΔCmwc-1* strain.
