# Supplementary figures and images for: Dynamic Genome-Wide Transcription Profiling and Direct Target Genes of CmWC-1 Reveal Hierarchical Light Signal Transduction in Cordyceps militaris
Source: J Fungi (Basel). 2022 Jun 11;8(6):624. doi: 10.3390/jof8060624 (PMC9225392; doi:10.3390/jof8060624)

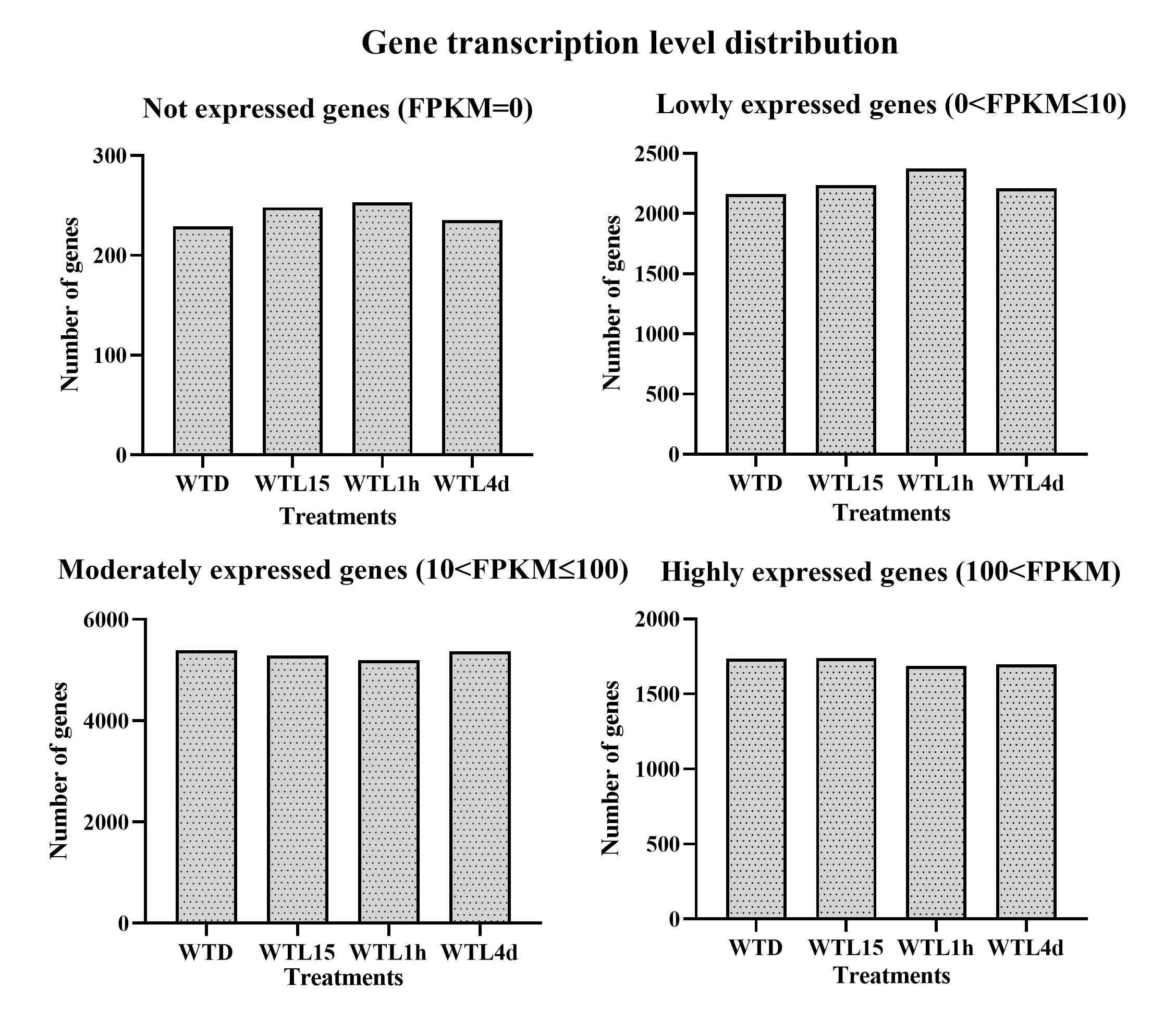

Supplement: Supplementary file 1 [file jof-08-00624-s001.zip › Figure S2.tif]

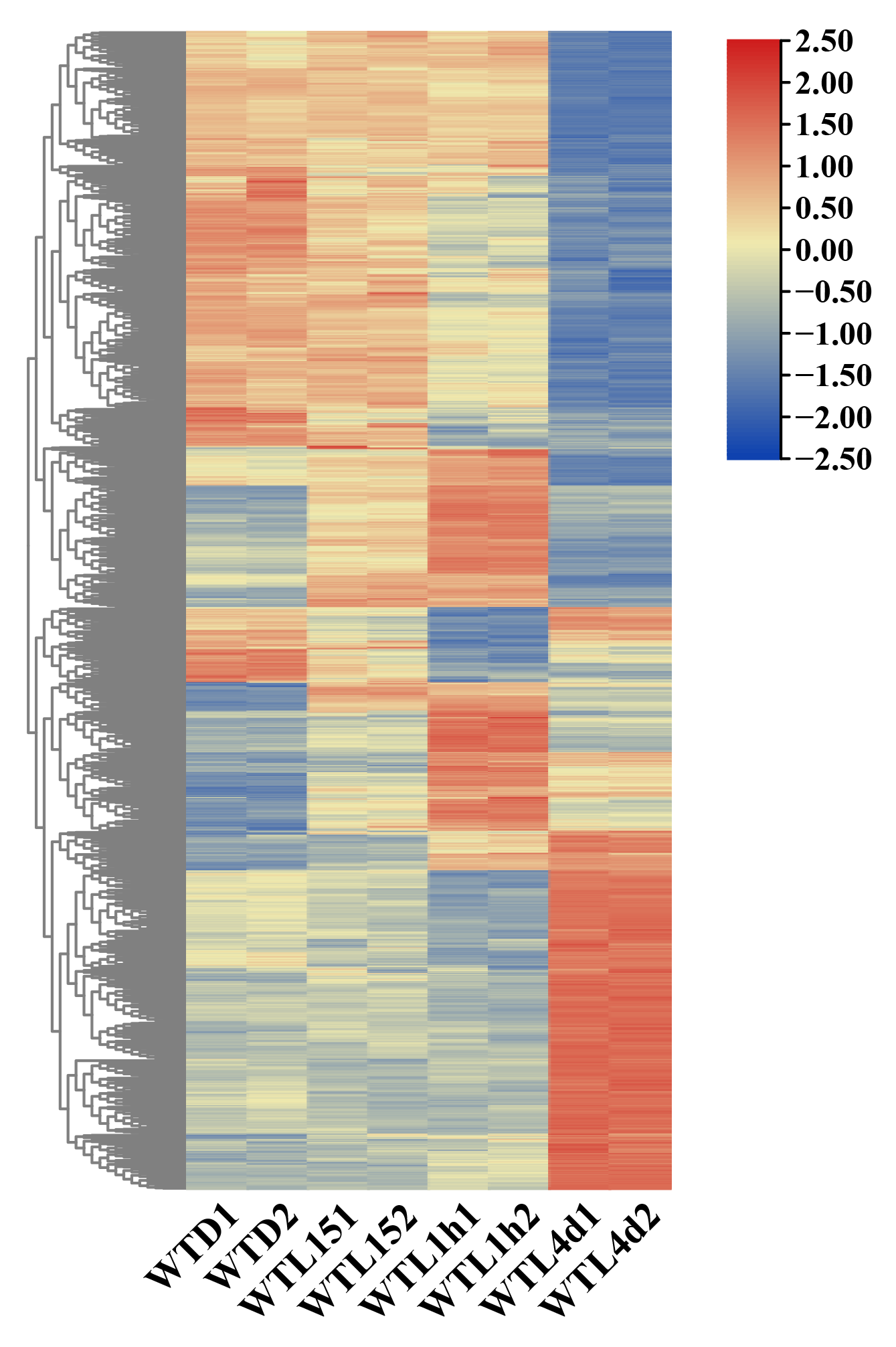

Supplement: Supplementary file 1 [file jof-08-00624-s001.zip › Figure S3.tif]

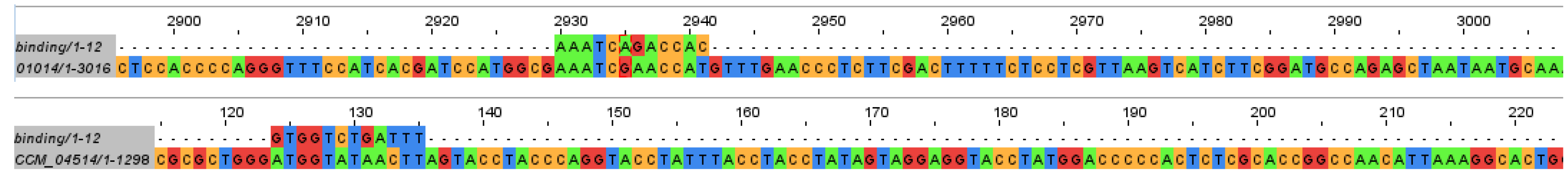

Supplement: Supplementary file 1 [file jof-08-00624-s001.zip › Figure S4.tif]
